# Transcriptional profiling of C57 and DBA strains of mice in the absence and presence of morphine

**DOI:** 10.1186/1471-2164-8-76

**Published:** 2007-03-16

**Authors:** Dorothy E Grice, Ilkka Reenilä, Pekka T Männistö, Andrew I Brooks, George G Smith, Greg T Golden, Joseph D Buxbaum, Wade H Berrettini

**Affiliations:** 1 Department of Psychiatry, University of Medicine and Dentistry of New Jersey/New Jersey Medical School, Newark, NJ, USA; 2 Division of Pharmacology and Toxicology, Faculty of Pharmacy, University of Helsinki, Finland; 3 Environmental and Occupational Health Science Institute, University of Medicine and Dentistry of New Jersey, Piscataway, NJ, USA; 4 Coatesville VA Medical Center, Coatesville, PA, USA; 5 Department of Psychiatry, Mount Sinai School of Medicine, New York, NY, USA; 6 Center for Neurobiology and Behavior, University of Pennsylvania, Philadelphia, PA, USA

## Abstract

**Background:**

The mouse C57BL/6 (C57) and DBA/2J (DBA) inbred strains differ substantially in many aspects of their response to drugs of abuse. The development of microarray analyses represents a genome-wide method for measuring differences across strains, focusing on expression differences. In the current study, we carried out microarray analysis in C57 and DBA mice in the nucleus accumbens of drug-naïve and morphine-treated animals.

**Results:**

We identified mRNAs with altered expression between the two strains. We validated the mRNA expression changes of several such mRNAs, including Gnb1, which has been observed to be regulated by several drugs of abuse. In addition, we validated alterations in the enzyme activity of one mRNA product, catechol-O-methyltransferase (Comt). Data mining of expression and behavioral data indicates that both Gnb1 and Comt expression correlate with aspects of drug response in C57/DBA recombinant inbred strains. Pathway analysis was carried out to identify pathways showing significant alterations as a result of treatment and/or due to strain differences. These analyses identified axon guidance genes, particularly the semaphorins, as showing altered expression in the presence of morphine, and plasticity genes as showing altered expression across strains. Pathway analysis of genes showing strain by treatment interaction suggest that the phosphatidylinositol signaling pathway may represent an important difference between the strains as related to morphine exposure.

**Conclusion:**

mRNAs with differing expression between the two strains could potentially contribute to strain-specific responses to drugs of abuse. One such mRNA is Comt and we hypothesize that altered expression of Comt may represent a potential mechanism for regulating the effect of, and response to, multiple substances of abuse. Similarly, a role for Gnb1 in responses to multiple drugs of abuse is supported by expression data from our study and from other studies. Finally, the data support a role for semaphorin signaling in morphine effects, and indicate that altered expression of genes involved in phosphatidylinositol signaling and plasticity might also affect the altered drug responses in the two strains.

## Background

The mouse C57 and DBA inbred strains differ substantially in many aspects of their response to drugs of abuse, including altered rates of voluntary drug consumption [1]. The C57 strain shows increased voluntary consumption of multiple drugs of abuse, including opiates, alcohol, cocaine, and amphetamine, which mirrors the poly-substance nature of some drug-seeking behavior in humans [2,3]. C57 strains of mice have been viewed as a good model for human drug-seeking behavior. It has been assumed that the poly-substance nature of drug-seeking in C57, as compared to DBA, is due, in part, to common mechanisms that operate irrespective of the drug of abuse. This assumption is fueled by studies showing that there are susceptibility factors that contribute to drug use irrespective of the substance, as well as susceptibility factors that are specific to each pharmacologic class [2-4].

Multiple quantitative trait locus analyses (QTL) have been carried in the C57 and DBA strains for both drug responses and drug-seeking behavior. QTL analyses for drug-seeking preferences to different classes of drugs might identify regions of the genome associated with drug-seeking across multiple pharmacological classes and, perhaps, more directly target common reward pathways for multiple substances. For morphine, the strongest evidence for a QTL underlying morphine preference is on the proximal part of chromosome 10, at the position of the mu opiate receptor [5-7]. In the case of preference for alcohol, the strongest QTL's are on chromosomes 2, 3, 4, and 9, as determined by meta-analyses of 8 studies of alcohol preference [8]. Further studies with other substances need to be carried out to determine regions of potential overlap. The development of microarray analyses represents an additional genome-wide method for measuring differences across strains, focusing on expression differences rather than on sequence differences in the genome. This is an additional approach to characterize variations between strains as a means of understanding molecular differences that might underlie strain variability in the effect of, and response to, multiple substances of abuse.

In the current study, we have carried out microarray analysis in C57 and DBA mice in the nucleus accumbens (NAC) of naïve and drug-treated animals. We identified a number of mRNAs with altered expression between the two strains. We validated the mRNA expression changes of several such mRNAs, as well as the protein activity of one mRNA product, catechol-O-methyltransferase (Comt). Data mining of expression and behavioral data indicates that Comt expression correlates with two aspects of drug response in C57/DBA recombinant inbred strains. Similarly, a role for Gnb1 in responses to multiple drugs of abuse is supported by data from our study and from other studies. Specific pathways were also identified that showed effects of treatment and/or strain. mRNAs with differing expression between the two strains could potentially contribute to strain-specific responses to drugs of abuse.

## Results

### Microarray analysis in the nucleus accumbens

We carried out microarray analysis in the dissected NAC from 9 vehicle-treated and 9 morphine-treated C57 and 9 DBA mice, using a pooling strategy to minimize dissection and other variations. Twelve groups of 3 NAC were hybridized to the Affymetrix MG_U74A microarray and the resultant data analyzed using dChip. We compared mRNA expression between strains, and across treatment groups.

The first analysis was gene oriented, and required a significant (>= 1.7) fold change and made use of t-testing to ascribe significance (P < 0.05) (see Methods). Comparing mRNA expression between C57 and DBA mice in the absence of morphine, we identified 67 mRNAs with altered expression, four (cathepsin e/Cathe, integrin beta 1 binding protein 1/Itgb1bp1, zinc finger protein 97/Zfp97, and the riken cdna 1110008e19 gene/glyoxalase 1/Glo1) with two different probe sets [see Additional file Table 1A]. Comparing mRNA expression between the two strains in the presence of morphine (via pellet) for 4 days, we identified 50 mRNAs with altered expression, four (Itgb1bp1, Glo1, guanine nucleotide binding protein, beta 1/Gnb1, and transcription elongation factor a (sii) 1/Tcea1) with two different probe sets [see Additional file Table 1B]. We also compared expression within each strain, contrasting expression in vehicle and morphine-treated mice [see Additional file Table 1C]. There were fewer morphine-responsive mRNAs within each of the two strains, when compared to the number of mRNAs that differed between strains. In the DBA strain, 8 mRNAs were identified as changed with an effect >= 1.7 fold in response to morphine and we did not identify any mRNAs showing a P < 0.05 and greater than >= 1.7 fold change in the C57 strain in response to morphine.

### Treatment-invariant mRNAs

It was of interest that genes showing greater expression changes in these first analyses were much more common across strains than within strains. Furthermore, there was a very significant proportion of mRNAs that showed altered expression between the two strains in both the drug-naïve and the morphine-treated groups [see Additional file Table 1, A, B]. We examined the correlation of the fold-expression changes of these treatment-invariant mRNAs under the two treatments and observed a linear relationship, with a high degree of correlation in the relative expression of these mRNAs (correlation coefficient of >0.98). These treatment-invariant mRNAs were of particular interest as they represent a molecular environment that is consistently different between the two strains, irrespective of treatment condition. They also provide evidence that the microarray studies are internally consistent.

### Two-way ANOVA

The presence of a large proportion of mRNAs that showed altered expression irrespective of treatment supported further analysis of the dataset, using two-way ANOVA. With such an approach, we could identify mRNAs showing main effects for strain, main effects for treatment, and interaction effects. We used more permissive criteria (no fold-effect requirement, but P < 0.01), so as to capture genes and pathways that demonstrated smaller but significant changes in expression. Running such analyses led to the identification of 973 genes that showed a main effect of strain, 185 genes showing a main effect of treatment, and 326 genes showing significant interaction effects [see Additional file Table 2A–C].

Gnb1 showed the most significant (P = 1.65 × 10^-10^) main effect for strain and additional treatment-invariant genes noted above (including Comt, P = 2.14 × 10^-7^) were also identified in this analysis. KEGG pathway analyses of genes showing main effect for strain identified clusters of genes associated with the ribosome, oxidative phosphorylation/ATP synthesis, long-term potentiation/long-term depression, and antigen processing and presentation [see Additional file Table 3A].

KEGG pathway analysis of genes showing a main effect of treatment [see Additional file Table 2B] identified two significant functional groups, including "axon guidance" [see Additional file Table 3B]. The axon guidance group of genes identified in these analyses were all related to the semaphorin pathway [see Additional file Table 2, B and Additional file Table 3B, as well as Discussion]. KEGG pathway analysis of genes showing significant strain by treatment interactions [see Additional file Table 2C] identified significant effects for genes in the focal adhesion pathway, particularly genes involved in phosphatidylinositol signaling system [see Additional file Table 2C and Additional file Table 3C, as well as Discussion].

### Validation of microarray analyses

We chose a selection of the mRNAs from the microarray studies for follow-up using quantitative polymerase chain reaction (QPCR) to validate the microarray signals. mRNAs were chosen to represent both the up-regulated and down-regulated mRNAs, to represent mRNAs showing more profound expression changes, to represent mRNAs with neuronal expression (analyzed by reviewing the expression of each probe set in mouse tissue [9]) and/or potentially relevant neuronal function, and based on other published studies on either varying expression in mouse strains or changes in response to drugs of abuse.

We were able to validate a majority of mRNAs for which we carried out QPCR, as shown in Table 1. Overall validation of the microarray results was reflected in the high correlation of the ratio of mRNA expression between the two strains as measured by microarray and by QPCR (Pearson's correlation coefficient, r>0.8, examining vehicle-treated mice). Furthermore, by ANOVA there was a significant (P < 0.05) main effect of strain with actin related protein 2/3 complex, subunit 5/Arpc5, Comt, Gnb1, phosphodiesterase 9a/Pde9a, phosphatidylinositol transfer protein, beta/Pitpnb, and secretory granule neuroendocrine protein 1, 7B2 protein/Sgne1, and a significant main effect of treatment with Arpc5, and Rcn. There was a trend level (P < 0.10) main effect of strain with Rcn, and a trend level main effect of treatment with Gabra2. Finally, there were significant interaction terms with Arpc5, ngfi-a binding protein 2/Nab2, and Rcn. Not all microarray results were unambiguously validated, as an mRNA showing great dys-regulation between the two strains by microarray (Pitpnb) did not show such profound alterations in expression by QPCR. Failure of validation can occur for several reasons, including differing probes used for the microarray and QPCR experiments (which can capture differential expression in splice variants), genomic differences between strains that alters the hybridization of the relevant regions of the probes, and false-positive expression changes.

### Comt levels and morphine

**Figure 1:**
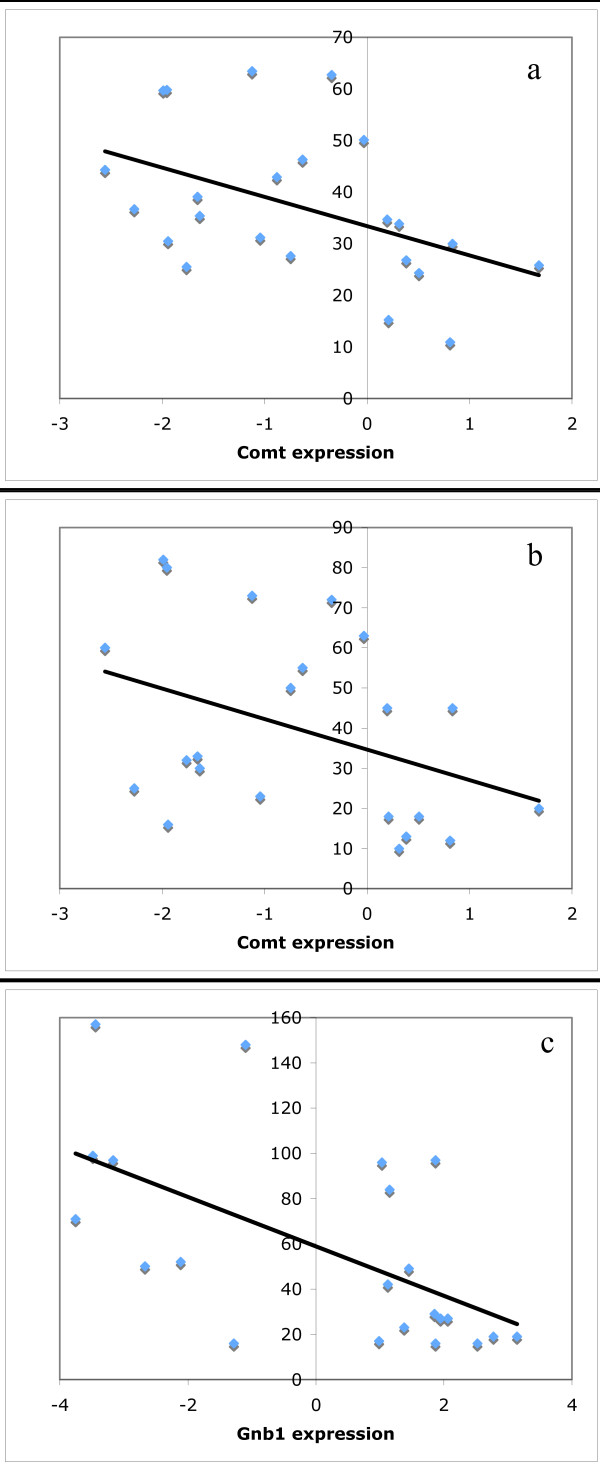
**Correlation of brain Comt and Gnb1 expression with morphine efficacy**. Comt and Gnb1 expression in BXD RI strains were correlated with the slope of the morphine dose-response curve (a, n = 23) (derived from [51]), with the analgesia observed with 16 mg/kg of morphine (b, n = 22) (analgesia is expressed as percent of maximum effect and is derived from Fig. 1 of [52]), or with morphine consumption in a two-bottle choice study (c, n = 22) [53] in the BXD RI strains. All expression and behavioural data can be found on the WebQTL database [13]. Comt expression was assessed using the single associated probe set (98535_at), while Gnb1 expression was assessed using probe set 94854_g_at. Rank order correlations were: Top, -0.535, P = 0.0076; middle, -0.470, P = 0.026; and, bottom, -0.586, P = 0.0035.

A recent report made the observation that individuals with a Met/Met genotype (associated with significantly decreased COMT activity) at the Val158Met polymorphism of the human *COMT *gene required a significantly reduced dose of morphine to achieve pain relief, as compared to individuals with the Val/Val genotype [10]. These observations are consistent with other studies implicating COMT activity with opioid response [11,12]. Since the DBA mice showed increased sensitivity to morphine compared to C57, as defined by the tail flick assay, and reduction in Comt expression and activity, we sought to determine the degree to which morphine sensitivity varied in C57/DBA recombinant inbred strains, as a function of Comt expression, making use of the WebQTL database [13]. We first noted that in those studies of whole brain homogenates, Comt expression was significantly decreased in DBA, as compared to C57, consistent with our studies in the NAC. We then examined the indices of morphine effect described above. Slope of the dose-response curve and the response to 16 mg/kg of morphine both correlated with levels of Comt expression (rank order correlations of -0.535, P = 0.0076, and -0.470, P = 0.026, respectively) (Figure 1). Comt expression did not show significant correlation with morphine consumption in a two-bottle choice paradigm (rank order correlation of 0.356, P = 0.104).

**Figure 2:**
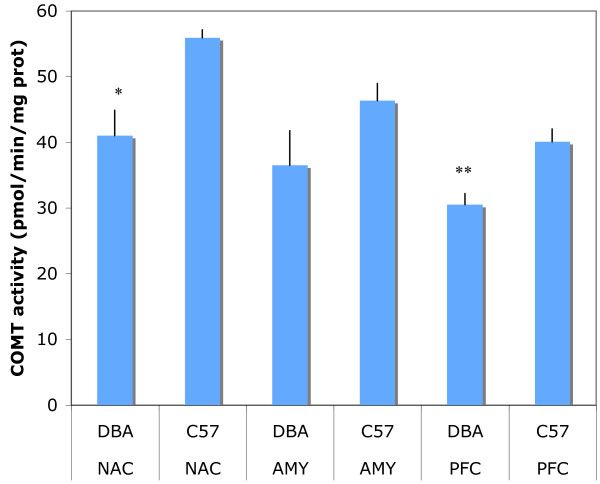
**Enzymatic assay of Comt activity in DBA and C57 strains**. Nucleus accumbens (NAC), amygdala (AMY), and frontal cortex (FC) were dissected from DBA and C57 mice, and Comt activity was determined in homogenates, as described in the methods. *, P = 0.01; **, P = 0.002.

The correlation of Comt expression with two indices of morphine effect suggested a possible relationship between these two measures in mice. As mRNA levels do not always correlate with protein levels and protein activity, we next sought to determine whether Comt activity differed in brain extracts from C57 and DBA mice. NAC, amygdala (AMY), and frontal cortex (FC) were dissected from DBA and C57 mice and Comt activity was then determined in homogenates. Comt activity was modestly, but significantly, reduced in NAC (p = 0.01) and FC (p = 0.002) of DBA mice, as compared to C57 mice (Figure 2).

## Discussion

We examined mRNA expression in the NAC in vehicle- or morphine-treated mice, comparing the C57 and DBA strains. The NAC is part of the mesocorticolimbic dopamine system which originates in the ventral tegmental area (VTA) and projects to the NAC, FC and other limbic areas [14]. The VTA and the NAC are two brain regions commonly thought to be involved in the reward system for drugs of abuse. In studies that compared gene expression changes induced by morphine treatment across multiple brain regions, the NAC shows some of the most profound changes in the total number of genes with altered expression in the presence of drug (e.g., [15]).

In the current study, we made use of an established and well-regarded analytical platform for analysis of microarrays. In our first analyses, we required reasonable levels of expression, a modest (>= 1.7) fold change, and a nominal P < 0.05. Median False-Discovery Rate (FDR) estimates were between ~1–4% for results contrasting the two strains, but were significantly higher (~20% for DBA) for contrasts within strains [see Additional file Table 1A–C]. Of importance to the current study is that Kerns et al. have recently compared mRNA expression in the NAC in control C57 and DBA animals [16]. Of the 67 mRNAs showing altered expression in our study of vehicle-treated mice, more than 20 (some of which, including Gnb1, Itgb1bp1, and Glo1, with two probes) were also observed to show altered expression (>1.7 fold change) in the Kerns study, in the same direction for the same probe sets, using different analytical methods (additional probe sets show altered expression of 1.4–1.7 fold change in the Kerns study, in the same direction). Furthermore, there was a significant agreement in relative mRNA expression across the two studies (correlation coefficient of ~0.8). This indicates that a significant proportion of mRNAs showing altered expression by microarrays can be reproduced across the two studies, irrespective of analytical approach. It also represents an important validation of such microarray studies.

While the Kerns study did not identify changes in Comt, our analysis of an independent dataset supported our results. In addition, microarray studies in the hippocampus of eight mouse strains demonstrated highest expression of Comt in C57, amongst all the strains (including DBA), and a significant inverse correlation of Comt expression with aggressive behaviors [17]. This same study identified a decrease in expression of Riken cDNA 1810037i17 in C57, compared with DBA, and a positive correlation of expression of this gene with aggressive behaviors. In the current study we observed increased expression of Comt in C57, as compared to DBA, and increased expression of 1810037I17Rik in DBA, with a very significant main effect of strain (P = 7.83 × 10^-7^). More generally, several genes showing altered expression in controls in our study (including Prdrx2, Comt, Zfp197, Gabra2, Gnb1, Sgne1, Glo1, and additional ESTs) also showed similar alterations in that study, when analyzed with both MAS5 and dChip, even though the focus was on an independent brain area.

Microarrays are very important tools for exploratory analyses, but have some important restrictions. Samples run on different platforms (e.g., [18]), or analyzed with different methods (e.g., [19-22]), can produce varying results. Quality of probes, probes directed at minor splice variants, and mis-annotation of probes can also lead to errors in conclusions (e.g., [23]). Furthermore, for a proportion of mRNAs with altered expression, there may not be a corresponding change in protein expression. For all of these reasons, validation by additional methods is critical. In recent QPCR analyses of striata from four strains of mice, Comt was found to be downregulated in DBA, as compared to C57 [24], consistent with the microarray, QPCR and enzyme activity results presented here. Similarly, Gabra2 was observed to be upregulated in DBA, as compared to C57, in that study as well.

A common mechanism for many drugs of abuse is the increase of dopamine in the pathway from the VTA through the NAC and to the FC [25]. In the case of morphine, binding of morphine to GABA interneurons reduces GABA release, thereby reducing the GABA-mediated inhibition of dopamine release in the NAC, with a net result of increasing dopamine levels. In the case of cocaine, blocking of dopamine re-uptake leads to increased dopamine levels, while in the presence of amphetamine, there is increased non-vesicular release of dopamine. Additional studies implicate these pathways in the responses to other substances of abuse. Elevation of dopamine would be inhibited by increased activity of Comt. We, therefore, can hypothesize that animals, or humans, with elevated Comt levels would experience reduced rewarding effects to drugs of abuse, while animals or humans with decreased levels of Comt may be more sensitive to certain drugs of abuse and perhaps even find them aversive. Alternatively, elevated Comt levels may lead to a hypo-dopamine state which drug taking might alleviate.

In several studies in human substance use, there has been evidence for an association of COMT genotype with substance use (reviewed in [26]). While these studies remain somewhat controversial, they are consistent with our hypotheses. These studies typically focused on the Val/Met variant in COMT. However, it may be that analysis of other variants within COMT, such as those associated with expression [27], may show stronger association with substance use.

A corollary of our hypotheses is that reduction of Comt activity, by the use of Comt inhibitors, may have two important effects. In the case of analgesia for severe pain, Comt inhibitors may reduce the levels of analgesics required, an hypothesis with some empirical support [10]. In addition, in instances of drug seeking behavior, Comt inhibitors may reduce drug seeking by reducing the "hypo-dopamine state," and/or by making the drug aversive or less rewarding. Studies on Comt in animals have previously demonstrated that non-specific COMT-inhibitors, when given in the presence of morphine, can be lethal [28,29]. While these results might suggest that there is an important molecular interaction between Comt and drugs of abuse, it is important to note that these studies were carried out with less specific, first generation COMT inhibitors.

Gnb1, one mRNA of a group of differentially expressed genes that we identified as also having enriched expression in the brain, showed significant correlation with morphine consumption in a two-bottle choice paradigm. Gnb1 was represented by three probe sets on the microarray that showed significant correlation with each other (rank order correlation 0.594–0.831, P 1.33 × 10-^11^-1.08 × 10^-04^). Correlation of the Gnb1 probe sets with morphine consumption showed rank order correlations of -0.586 (P = 0.0035; Fig. 1), -0.458 (P = 0.031), and -0.381 (P = 0.080), for probe sets 94854_g_at, 94853_at, and 97458_at, respectively. Altered expression of Gnb1 was confirmed by QPCR. All three probe sets of Gnb1 were observed to have decreased expression in the DBA strain under control conditions in the NAC in an independent study [16]. Moreover, in that study Gnb1 also showed decreased expression in the FC in the DBA strain. Finally, Gnb1 (examining probe set 94853_at) was previously shown to have a five-fold range of expression in BXD recombinant inbred strains, with a heritability of 75%, which is near the maximum observed for any transcript [30]. Gnb1 is a beta-subunit for the heterotrimeric guanine nucleotide-binding proteins, and is also known to play an important role in the visual system.

In our studies, Gnb1 expression, as measured by QPCR, showed modestly increased (~50%) expression in both strains in the presence of morphine. Interestingly, Gnb1 expression has been shown to be up-regulated in the NAC in response to cocaine administration in mouse [31] and rat [32], and in response to amphetamine [31] and methamphetamine [33] treatment in the mouse. Antisense disruption of Gnb1 expression blocks cocaine-induced sensitization, but without affecting the acute responses to cocaine [31]. In a recent study examining genes that show altered expression four hours after naltrexone-induced morphine withdrawal, Gnb1 was downregulated in the locus coeruleus (LC) of C57 mice [34]. The LC is the primary site of noradrenergic neurons in the brain, and sends projections throughout the mesocorticolimbic dopamine system, including to the NAC, VTA, and FC, and is a mediator of physical dependence on opiates [14]. Gnb1 maps to murine chromosome 4 (at 15 Mb) and GNB1 maps to human chromosome 1p36.33. A role for Gnb1 expression in responses to multiple drugs of abuse is supported by the expression data.

By the nature of our experimental design, we were able to consider strain and treatment effects in the same study. Such an approach has not been undertaking previously. There were multiple genes showing main effects of strain with much more modest numbers of genes showing main effects of treatment or strain by treatment interactions. One facet of our results is consistent with the hypothesis that an important determinant underlying the differences in morphine response across strains lies in genes that show a main effect of strain and are not necessarily regulated by morphine. Such genes include Gnb1 and Comt, as discussed in detail above. Other genes showing main effects of strain are genes important in plasticity, including those defined in the KEGG pathways of long-term potentiation (LTP) and long-term depression (LTD). Genes in these pathways that show a main effect of strain by ANOVA include an ionotropic glutamate receptor subunit (NMDA2B), two isoforms of calcium/calmodulin-dependent protein kinase II (CaMKII), phosphatases [protein phosphatase 3/calcineurin (CaN) and protein phosphatase 2 (formerly 2A)], an inositol 1,4,5-triphosphate receptor (IP3R), ras proteins (Ras), and a mitogen activated kinase in the LTP pathway. In the LTD pathway, KEGG analysis identified several guanine nucleotide binding protein (G protein) subunits, phospholipase A2, the mitogen activated kinase, protein phosphatase 2 and IP3R identified above, as well as insulin-like growth factor 1. These plasticity genes may contribute to multiple differences between the strains, including to drug responses. In this context, the genes identified in the long-term potentiation pathway are interesting. The NMDA receptor modulates intracellular calcium levels, as does IP3R. This in turn can modulate calcium dependent protein phosphatases, kinases and ras proteins, followed by changes in activity of mitogen activated kinases. Expression of representatives of all of these classes of enzymes are altered across the two strains, and may thereby contribute to altered drug dependence (e.g., [35]).

Genes showing a main effect of treatment are related to morphine response, irrespective of strain. These genes fell into a diverse group. KEGG analysis identified two significant groups, identified as "adipocytokine signaling pathway" and "axon guidance." The axon guidance group of genes identified in KEGG analysis were all related to the semaphorin pathway, with semaphorins 3F, 4B, 6C, 6D, and 7A showing treatment effects. A potential semaphorin receptor, plexin A3, was also identified as showing a main effect of treatment. This is an interesting finding in light of a recent study looking at the effects of cocaine on semaphorins and other axon guidance molecules in adult rat [36]. Many of the semaphorins, including those above, showed significant expression changes in response to cocaine. Although that study made use of cocaine, it may be that changes in axon guidance molecules are a frequent response to drugs of abuse (see [37]).

Another aspect of the drug by strain analysis allows for looking at genes that are differentially regulated across strains in response to morphine. These genes may also contribute to altered drug response between C57 and DBA mice. KEGG analysis identified genes within the focal adhesion pathway as showing drug by strain interactions in ANOVA. Several extracellular matrix (ECM) genes (collagen and laminins) were identified, as were two integrins (beta5 and alpha2), which can bind to ECM proteins. Genes downstream of the integrins, including phosphatidylinositol 3-kinase (PI3K), were altered, as well as genes regulated by the phosphatidylinositol signaling system (3-phosphoinositide dependent protein kinase and Vav oncogene). These results suggest that the phosphatidylinositol signaling system is of interest in individual variations in drug responses, especially considering that there is recent evidence for a role for this system in morphine withdrawal and cocaine sensitization [38,39].

QTL analysis, as a means for understanding molecular differences across strains, has proven more difficult than anticipated. As elaborated in a recent review [40], over the past 15 years, there have been more than 2,000 QTLs that have identified in crosses between inbred strains of mice, and 700 QTLs found in crosses between inbred strains of rats. However, only about 20 genes have been identified from these QTL analyses, many of which have LOD scores of over 10. The overall success rate of less than 1% for identifying genes underlying QTLs has been explained by various means, and likely reflects a hidden complexity in the QTL analyses. The use of microarrays to supplement QTL analyses may simplify the identification of quantitative trait genes (QTGs) that underlie QTLs and may also identify additional genes that have not been mapped by QTL analysis. It is of note that QTL analyses of drug-related behavior in mice will not necessarily identify the Comt, Gnb1 or other loci. However, it is important to appreciate that genetic factors modulating expression of a given gene can localize either to the gene (cis) or to other loci in the genome (trans). For example, expression QTL (eQTL) analysis of murine Comt, using the UTHSC website [41] demonstrate suggestive (>2.27) LOD scores near the Comt locus (at 20 Mb), but also at murine chromosome 10 (105–110 Mb).

## Conclusion

In summary, there are multiple mRNAs that show differential expression between the C57 and DBA strains. Of them, we highlight Comt and Gnb1 as potentially contributing to altered drug-related behaviors in C57 as compared to DBA mice. We suggest that inhibition of Comt by pharmacological methods may reduce desire for drugs and perhaps cause them to be more effective in clinical settings and/or less rewarding in non-clinical settings. Pathway analysis highlights important differences between the two strains with regards to plasticity (LTP and LTD), which may contribute to some of the behavioral differences across the strains, including those associated with drug-related behaviors. Furthermore, pathway analysis supports a role for semaphorin signaling in morphine effects, and indicates that altered expression of genes involved in phosphatidylinositol signaling and plasticity might also affect the altered drug responses in the two strains.

## Methods

### Mice

Mice were obtained from the Jackson Laboratory. Animals were male, 8–12 weeks old, with a weight range of 18 to 28 grams. Animals underwent surgery for subcutaneous implantation of either a control or morphine pellet (either the "Placebo to Morphine Base Implant Pellet" or the 25 mg "Morphine Base Implant Pellet," both the kind gift of the National Institute on Drug Abuse) on the dorsal aspect of the neck. The composition of the pellets was identical (microcrystalline cellulose, 149 mg; magnesium stearate, 1.5 mg; colloidal silicon dioxide, 2.5 mg) except for the addition of 25 mg of purified morphine. While the animal was under anesthesia (ketamine-HCl and xylazine), a 1/4-inch slit was made in the skin, the pellet inserted and the area closed with a surgical metal staple. Morphine-treated mice from both strains demonstrated dorsiflexion of the tail (Straub tail). Consistent with previous results [42], there appeared to be increased home cage locomotion in morphine-treated C57 mice. We implanted the pellet on Day 1 and sacrificed on Day 5. Euthanasia was carried out using CO_2 _with cervical dislocation, and the brains rapidly dissected. Thick sections (500–600 μm) were sliced on a Vibratome, and the structures (frontal cortex, amygdala, and nucleus accumbens) were dissected from these sections under a dissecting scope, using delineations and stereotaxic coordinates from a mouse brain atlas [43]. The caudal aspect of frontal cortex was defined by the level at which the genu of the cortex collosum became visible. The animal protocol was approved by the Coatesville VA Medical Center IACUC.

### Microarrays

Nucleus accumbens was dissected from nine C57 and nine DBA male mice. Tissue was then pooled into groups of three samples, as a means of minimizing variation due to dissection. RNA was purified using Trizol reagent (Gibco), and RNA quality was confirmed by 260/280 ratio, and by subsequent analysis on an Agilent Bioanalyzer. Between 1 and 10 μg of total RNA from each sample was used to generate a high fidelity cDNA, which is modified at the 3' end to contain an initiation site for T7 RNA polymerase. Upon completion of cDNA synthesis 1 μg of product was used in an in vitro transcription (IVT) reaction that contains biotinylated UTP and CTP utilized for detection following hybridization to the oligonucleotide microarray. 20 μg of full-length cRNA was fragmented in 200 mM Tris-actetate (pH 8.1), 500 mM KOAc and 150 mM MgOAc at 94C for 35 minutes. Following fragmentation all components generated throughout the processing procedure (cDNA, full-length cRNA, and fragmented cRNA) were analyzed by electrophoresis using the Agilent Bioanalyzer 2100 to assess the appropriate size distribution prior to microarray hybridization. Detailed protocols for sample preparation using the Affymetrix labeling protocols can be found at [44].

Samples were subjected to hybridization against the Affymetrix U74A high-density oligonucleotide array. Hybridization, staining and washing of all arrays was performed in the Affymetrix fluidics module as per the manufacturer's protocol. Streptavidin phycroerythrin stain (SAPE, Molecular Probes) was the fluorescent conjugate used to detect hybridized target sequences. The detection and quantitation of target hybridization was performed with a GeneArray Scanner (Hewlett Packard/Affymetrix) set to scan each array twice at a factory set PMT level and resolution. Arrays were assessed for "array performance" prior to data analysis. This process involves the statistical analysis of control transcripts that are spiked into the samples and the hybridization cocktail to assess consistency of signal quality. Samples met a minimum set of standards in order for the array (and the sample) to be included in downstream analysis. All arrays had 3'/5' ratios (for BACTIN and GAPDH) of less than 2.5, indicating a lack of bias in the RT and cRNA amplification process. All arrays achieved a % present call rate of 45% or greater.

The dChip algorithm (version 1.3) was used to analyze all microarray data [45]. dChip takes advantage of the high degree of standardization of oligonucleotide arrays to deal with two recurrent problems with oligonucleotide arrays. The first problem is the large differences that often exist in results obtained with different probes to the same mRNA. The second is the inevitable presence of large or small regions of contamination on any given array. Because the synthesis of the arrays is so standardized, the behavior of each probe is highly reproducible and predictable. Thus using modeling and appropriate analysis, the relative reliability of a probe can be estimated and a weighted index of expression can be determined (a model-based expression index, or MBEI). The MBEI has a standard error associated with it as a measure of accuracy. Furthermore, probe sets within an mRNA that, for a particular experiment, show profiles that deviate significantly from the typical profile, can be automatically flagged as likely reflecting contamination or cross-hybridization. As a primary analysis, we used Invariant Set Normalization and PM-MM modeling [45], with a threshold of 1.7-fold change, a nominal p value < 0.05, a percent call of >80%, and an absolute differences in expression of > 50 (to eliminate mRNAs that were expressed at near-background level) to choose genes for further analysis. The nominal p value was determined using dChip (which down-weights unreliable expression data), testing whether the mean difference between two groups equals to zero by the unpaired t-test. False discovery rates (FDR) for each pair of datasets contrasted were empirically estimated with 200 random permutations of the data.

Given the clear evidence for treatment-invariant genes (see Results) in these first analyses, we also carried out two-way analysis of variance (ANOVA) using dChip, identifying genes with a main effect of strain or treatment, or with an interaction effect. Here the requirement was simply a P < 0.01. This less conservative analysis identified many more genes, and Gene Ontology as well as Kyoto Encyclopedia of Genes and Genomes [46] pathway analysis was carried out to identify classes of genes and pathways that were particularly affected.

Annotation, Gene Ontology, and KEGG pathway analyses were carried out with DAVID (Database for Annotation, Visualization and Integrated Discovery 2007, [47]). Raw microarray data from this study is available on the NCBI GEO website.

### Real time quantitative RT-PCR (Q-PCR)

Nucleus accumbens was dissected from male C57 and DBA mice that had been treated for 4 days with either a control or a morphine pellet. mRNA expression was examined using Taqman chemistry. We acquired commercially validated assays from ABI for gene expression analysis. The assay ID's for the genes interrogated are as follows: Arpc5, Mm00848130; Comt, Mm005714377; Gnb1, Mm00515002; Nab2, Mm00476267; Pde9a, Mm00501039; Pitpnb, Mm01205388; Sgne1, Mm00486077; and Rcn, Mm00485644. The following dye combinations for probe generation were used for detection and data normalization: FAM (for the mRNAs of interest), HEX (for GAPDH, as a normalizer mRNA) and BHQ1 (non-fluorescent quencher) and ROX (reference). GAPDH was selected as a reference mRNA for comparative analysis based on its behavior in the microarray data set, showing no signal value differences in different strains. Following probe and primer optimization all cDNA's were diluted and used in a 10 μl PCR reaction containing: 5 μl of ABI 2x Universal Master Mix, 1.25 μl of each forward and reverse primers (final concentrations ranging from 200–900 nM depending on the primer set), 1 μl of probe (final concentrations ranging from 50–200 nM depending on the probe/primer set) and RNAase/DNAase free water. All reactions were performed in triplicate. Reactions were run in an ABI 7900 with the following cycle parameters: 1 cycle of 50°C (2 min) followed by 95°C (10 min.), 40 cycles of 95°C (15 sec) followed by 60°C (1 min). Data were collected at every temperature phase during every cycle, and analyzed using the Sequence Detection Software (ABI, Foster City CA). Relative quantitation using the comparative threshold cycle (CT) method was performed in Microsoft Excel (ABI Technote #2: Relative Gene Expression Quantitation). Real-time quantitative PCR P-values were calculated using two-way ANOVA carried out independently for each gene target.

### Enzymatic analysis of Comt

Nucleus accumbens, amygdala, and frontal cortex were dissected from male C57 and DBA mice. Assay of Comt was carried out as described earlier [48], with 500 μM substrate, instead of 240 μM.

### Analysis of mRNA expression as a function of morphine response

We made use of the April 05 data freeze, providing estimates of mRNA expression in brains of C57BL/6JXDBA/2J recombinant inbred mice, generated at the University of Tennessee Health Science Center (UTHSC) [41]. In order to be consistent throughout our own studies, which made use of the Affymetrix U74Av2 microarrays we made use of data measured using the same microarrays. At UTHSC, over 300 brain samples from 35 strains were hybridized in small pools (n = 3) to 100 arrays. Data were processed using the S-score software [49,50], although other analysis methods produced equivalent results. The S-score method centers expression of every probe set at 0. The signal values are therefore strain deviations in Z score units from the grand mean based on all arrays. For phenotype data, we made use of the data from published studies that characterized the morphine analgesia dose-response curve slope, hot plate analgesia in response to 16 mg/kg morphine, or morphine consumption in a two-bottle choice paradigm [51,52].

## Authors' contributions

DEG supervised or carried out the molecular studies, data analysis, and drafted the manuscript. IR and PTM carried out the Comt assays in tissue samples. AIB and the University of Rochester Medical Center Functional Genomics Center carried out the microarray analyses. GGS and GTG treated the mice and carried out all dissections. JDB participated in the molecular studies and data analysis, and participated in drafting the manuscript. WHB participated in the design of the study, and the supervision of the molecular studies and data analysis. DEG, IR, PTM, AIB, GGS, JDB, and WHB contributed to and approved the final manuscript.

## Supplementary Material

Additional file 1 STable1. The data provided represent genes showing differential expression. A. Genes showing differential expression between vehicle-treated C57 and DBA strains. B. Genes showing differential expression between morphine-treated C57 and DBA strains. C. Genes showing differential expression between vehicle and morphine-treated DBA. Click here for file

Additional file 2 STable2. The data provided represent genes showing differential expression using ANOVA. A. Genes showing main effect of strain. B. Genes showing main effect of treatment. C. Genes showing a strain by treatment interaction. Click here for file

Additional file 3 STable3. The data provided represent KEGG pathway analysis for showing differential expression using ANOVA. A. Genes showing main effect of strain. B. Genes showing main effect of treatment. C. Genes showing a strain by treatment interaction. Click here for file

## Figures and Tables

**Table 1:** QPCR validation analysis of select neuronal mRNAs.

	C57 Control	C57 Morphine	DBA Control	DBA Morphine
Arpc5*+!	1.00 0.13	1.42 ± 0.19	5.48 ± 0.38	13.61 ± 0.23
Comt*	1.00 ± 0.09	1.68 ± 0.39	0.71 ± 0.20	0.64 ± 0.05
Gabra2	1.00 ± 0.20	2.38 ± 0.34	3.99 ± 0.40	2.67 ± 0.62
Gnb1*	1.00 ± 0.10	1.46 ± 0.36	7.85 ± 0.22	12.13 ± 0.12
Nab2!	1.00 ± 0.10	1.28 ± 0.33	1.25 ± 0.36	0.41 ± 0.05
Pde9a*	1.00 ± 0.05	1.03 ± 0.06	2.04 ± 0.41	2.66 ± 0.31
Pitpnb*	1.00 ± 0.07	0.94 ± 0.05	0.34 ± 0.42	0.37 ± 0.15
Rcn+!	1.00 ± 0.03	6.18 ± 0.92	3.27 ± 0.81	1.47 ± 0.27
Sgne1*	1.00 ± 0.12	0.99 ± 0.07	3.88 ± 0.32	2.59 ± 0.19

Nucleus accumbens (NAC) from vehicle or morphine (4 d) treated C57 or DBA mice were dissected and used for QPCR analysis. Eight neuronal mRNAs are profiled above, showing for each mRNAs the expression in C57 control, C57 with morphine, DBA control, and DBA with morphine, always normalized to the average expression in C57 control. Data represent means ± SEM of expression normalized to the average expression in C57 under control conditions. *, P < 0.05 for main effect of strain; +, P < 0.05 for main effect of treatment; !, P < 0.05 for interaction.
